# Signet ring adenocarcinoma of the breast diagnosed endoscopically

**DOI:** 10.1016/j.igie.2025.08.002

**Published:** 2025-08-22

**Authors:** Michael Hayes, John Ahn, Cameron Schauer

**Affiliations:** Te Whatu Ora Waitemata, Auckland, New Zealand

A 69-year-old woman with no known medical history presented with 4 months of lethargy, fevers, and increasing abdominal girth. Initial examination revealed normal vital signs with ascites and axillary adenopathy. Blood test results demonstrated normocytic anemia with a hemoglobin of 8.0 g/dL (12.1-15.1 g/dL), an elevated carcinoembryonic antigen (CEA) level of 127.6 μg/L (<5 μg/L), and a cancer antigen 15-3 level of 883 U/mL (<30 U/mL). Contrast-enhanced computed tomography of the abdomen was remarkable for ascites, widespread adenopathy, and sclerotic and lytic bone lesions.

Fine-needle aspiration of an axillary node and ascitic fluid drainage were nondiagnostic. Colonoscopy, prompted by the elevated CEA, revealed widespread nodules with central dimples scattered throughout the transverse colon mucosa (Fig. 1A and B). Biopsy specimens of the nodules were obtained. Immunohistochemistry staining identified poorly differentiated adenocarcinoma with signet ring cell morphology, consistent with metastatic lobular breast carcinoma (Fig. 1C). Since diagnosis, the patient has responded favorably to palliative letrozole therapy.

**Figure 1:**
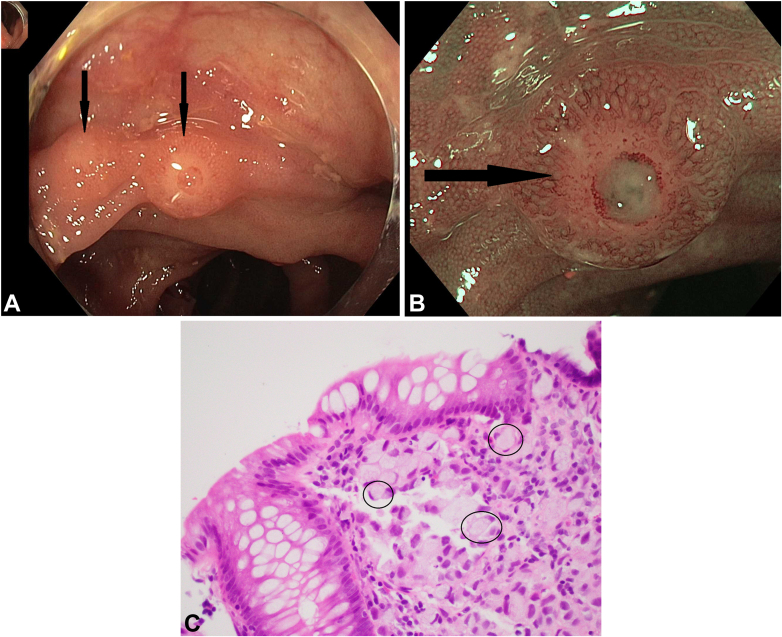
**A**, Endoscopic view of transverse colon nodular lesions under white light with nodules identified (*arrows*). **B**, Transverse colon nodule (*arrow*) visualized with narrow-band imaging. **C**, Hematoxylin and eosin, original magnification ×40 demonstrating multiple signet ring cells with a selection of signet ring cells *circled*.

Breast cancer with colonic metastasis is rare, with few cases existing in literature. Otsuka et al^1^ presented a case series of 3 patients in 2023. Bolzacchini et al^2^ described this disease in a systemic literature review as rare, but an important entity to consider in patients with lobular breast carcinoma. Breast metastases to the colon are most frequently encountered in lobular breast carcinoma.^1^ The endoscopic appearances are variable, ranging from focal abnormalities to obstructing lesions.^3^ The propensity for these tumors to metastasize to unusual sites, including the colon, is presumed to be due to the loss of E-cadherin, a protein required for cellular adhesion.^4^ Prognosis for patients is poor.^1^ Clinicians should consider this rare entity when encountering diffuse colonic nodules during colonoscopy.

## Patient consent

The patient in this article has given written informed consent to publication of their case details.

## Disclosure

The author discloses no financial relationships.
